# Brain age prediction model based on electroencephalogram signal and its application in children with autism spectrum disorders

**DOI:** 10.3389/fneur.2025.1605291

**Published:** 2025-06-18

**Authors:** Yi Ju, Tong Zhao, Zaifen Gao, Wenguang Hu, Jiejian Luo, Nian Cheng, Chunli Liu, Yuwu Jiang, Bo Hong, Taoyun Ji, Yuxiang Yan

**Affiliations:** ^1^First Hospital, Peking University, Beijing, China; ^2^Gnosis Healthineer Co., Ltd., Beijing, China; ^3^Jinan Children’s Hospital, Jinan, China; ^4^Chengdu Women and Children’s Central Hospital, Chengdu, China; ^5^Department of Biomedical Engineering, School of Medicine, Tsinghua University, Beijing, China; ^6^Ningxia Hui Autonomous Region Maternal and Child Health Hospital, Yinchuan, China

**Keywords:** brain AGE gap estimate (Brain AGE), autism spectrum disorder, EEG, Gate Recurrent Unit (GRU) neural network approach, pediatric

## Abstract

**Background:**

There is a lack of objective biomarkers for brain developmental abnormalities of autism spectrum disorder (ASD). We used EEG and deep learning to conduct a brain aging study in ASD.

**Methods:**

(1) A total of 659 healthy children and 98 ASD patients were retrospectively recruited. (2) An Auto-EEG-Brain AGE prediction model based on the Gate Recurrent Unit (GRU) neural network method was constructed. (3) Using the constructed model, we evaluated the difference between the brain age of ASD and that of healthy controls, and assessed the feasibility in the clinical assessment of ASD.

**Results:**

(1) The correlation coefficient (*r*-value) of the model exceeded 0.8 at the whole-brain level, with the highest value reaching 0.91. (2) *r*-values of the ASD group amounted to 0.76 at the level of the whole brain and ranged from 0.66 to 0.7 at the level of the sub-brain regions. The mean value of the brain age gap estimate (Brain AGE) in the whole brain is 0.76 years; in the sub-brain model, was 0.64–1.18 years.

**Conclusion:**

We constructed the EEG-Brain AGE prediction model, which can identify an individual’s brain development and be used as a biomarker for the brain development assessment in ASD.

## Introduction

Autism spectrum disorder (ASD) is a group of heterogeneous neurodevelopmental disorders, characterized by clinical manifestations such as social communication deficits, repetitive stereotyped behaviors, narrow interests, and sensory abnormalities. ASD has its inherent pattern of brain developmental abnormalities.

The brain is the most complex organ in the human body, and its development is highly coordinated, orderly, progressive, and region-specific, involving multiple aspects, including morphology, structure, and function (1). Therefore, precisely analyzing the brain development abnormality of ASD can help accurately assess individuals. Chronological age (CA) is an objective datum calculated based on an individual’s date of birth. Brain age (BA) is an objective biomarker that can visually measure the level of brain development and the degree of aging, aiming to identify the markers for pathological brain development and the aging process with the help of physiological BA trajectories (2). Brain age gap estimate (Brain AGE) is an important parameter that means the difference between the brain age indicated by the multivariate effect of the age-related information of the whole brain and the actual age of the individual. Brain AGE is an important parameter that can help to estimate the extent to which brain development deviates from the normal trajectory in a given state (3–5). Up to now, BA and BA prediction models have already been applied in schizophrenia (6, 7), bipolar disorder (8), and other disorders, but little research has been done in ASD. According to the previous study, MRI is the most commonly used tool for BA prediction (9). For example, Brown et al. (10) utilized multidimensional brain features to predict BA.

The transmission of EEG signals is an essential aspect of brain functions. Changes in the patterns of EEG activity are closely associated with brain development. However, research on BA using EEG is currently in its initial stage. The parameters of EEG can be potential biomarkers for ASD.

Therefore, based on the need for objective biomarkers for ASD and the advancement of EEG technology, as well as the deep learning field, we conducted this study. The study consists of two parts: firstly, an Auto-EEG-Brain AGE prediction model based on the GRU neural network approach was constructed. The correlation between the model’s predicted BA and CA was verified, indicating that the BA prediction model can achieve relatively accurate BA predictions for typically developing children. Secondly, the Auto-EEG-Brain AGE prediction model was used to quantify and analyze the statistical significance of individual brain development levels in ASD patients and their paired healthy children. The results showed that Brain AGE in ASD children was significantly higher across all brain regions compared to typically developing children. The aim is to demonstrate that BA and Brain AGE based on EEG signals can be used as a relatively objective and novel biomarker for diagnosing and assessing ASD.

## Methods

### Participants

Retrospective data from healthy children and ASD patients were collected from the electronic medical record systems and the electrophysiology department across multiple hospitals.

The inclusion criteria for healthy children were as follows: (1) age between 0 and 18 years; (2) normal EEG of healthy children. Exclusion criteria included: (1) incomplete medical history, missing critical data such as scale assessment results or EEG data; (2) history or current diagnosis of neurological or psychiatric disorders; (3) history or current use of neuro-psychiatric medications.

The inclusion criteria for ASD patients were as follows: (1) age between 0 and 18 years; (2) diagnosis of ASD according to the Diagnostic and Statistical Manual of Mental Disorders-Fifth Edition (*DSM-5*) and evaluated through the Autism Diagnostic Observation Schedule, 2nd (ADOS-2). Exclusion criteria included: (1) incomplete medical history, missing critical data such as scale assessment results or EEG data; (2) severe organic brain dysfunctions; (3) concurrent epilepsy and cerebral palsy; (4) serious chronic disease.

A total of 98 children with ASD were enrolled in our study. The age at diagnosis ranged from 2.17 to 8.42 years, with a mean age of 3.85 ± 1.83 years. The age at EEG recording ranged from 2.98 to 13.17 years, with a mean age of 5.25 ± 1.97 years.

### EEG data and pre-processing

**Figure 1:**
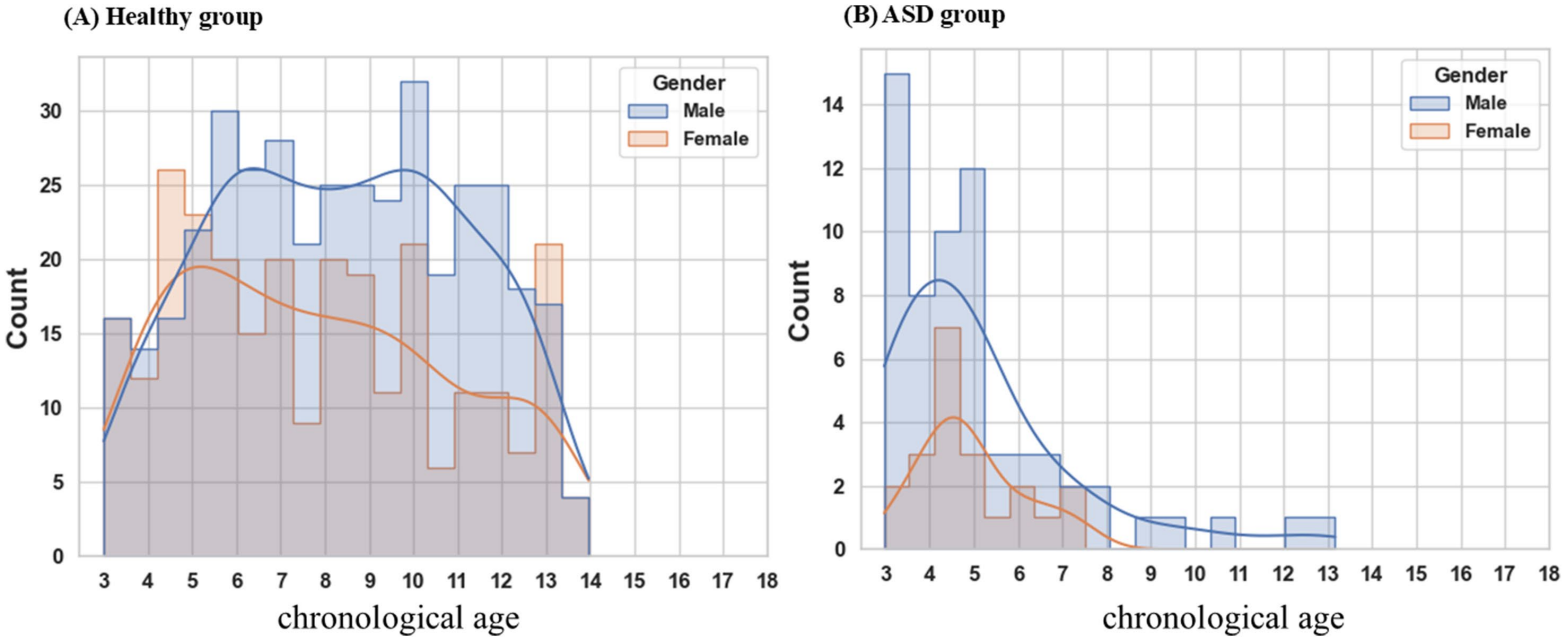
Age distribution by gender. **(A)** The healthy cohort encompasses individuals aged 3 to 14 years, exhibiting a male-to-female ratio of 1.42. **(B)** ASD patients range in age from 3 to 8 years, with a male-to-female ratio of 3.08. ASD autism spectrum disorder.

Resting-state EEG data were recorded using the Nihon Kohden EEG-1200 system (Tokyo, Japan), following the international 10–20 system for placing 19 scalp electrodes and two auricular electrodes. The sampling frequency exceeded 500 Hz. Impedances for the EEG were kept below 5 kΩ. The EEG recording duration for each participant ranged from 2 to 4 h, encompassing both wakefulness and sleep periods. We extracted 20 min of raw awake-state EEG data for analytical processing. The study comprised a comprehensive dataset of 772 EEG recordings from 659 healthy children (387 males, 272 females; ratio = 1.42), aged 3 to 14 years (Figure 1A). Additionally, the current research enrolled 98 EEG datasets from 98 patients (74 males, 24 females; ratio = 3.08) diagnosed with ASD (Figure 1B). The EEG dataset of the healthy children was stratified into distinct training, validation, and testing subsets, adhering to a partitioning ratio of 8:1:1, respectively. Each EEG recording from a healthy child was exclusively allocated to one of these subsets. The training subset comprised 600 EEG recordings obtained from 529 healthy children, constituting the dataset for model training.

EEG data preprocessing, including artifact removal and signal segmentation, was performed as follows. First, the EEG recordings were down-sampled to 200 Hz, followed by a common average referencing and applying a band-pass filter with cutoff frequencies set at 1 Hz and 70 Hz. Second, signal amplitudes exceeding 200 μV were identified as artifacts. Third, EEG recordings during wakefulness were segmented into intervals using a 5-min window with a step length of 30 s. Segments with an artifact proportion less than 0.30 were retained. Finally, the short-time Fourier transform (STFT) was performed on discrete 5-min epochs using a Hanning window with a 4-s temporal duration (800 sampling points at 200 Hz sampling rate) and 50% overlap (2-s overlap interval). The resultant time-frequency representations were subjected to spectral truncation, retaining only the 1–40 Hz frequency band, to optimize computational efficiency while capturing physiologically relevant neural oscillations. These segments were then utilized to construct the Auto-EEG-Brain AGE prediction model. The data preprocessing was implemented using the MNE (version 1.0) in Python (version 3.7.5).

### Model architecture

**Figure 2:**
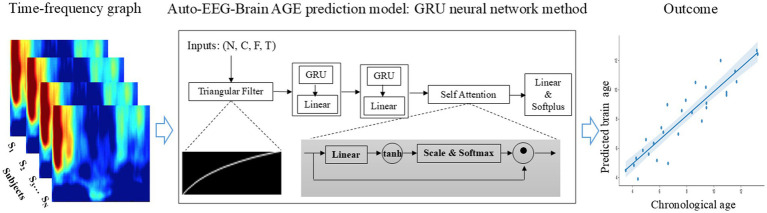
End-to-end brain age prediction procedure. The input is the time-frequency graph of 19 EEG electrodes from the 10-20 system of a 5-minute EEG segment. The Auto-EEG-Brain AGE prediction model includes a trainable triangular filter layer, two unidirectional GRU layers, an attention layer, and a Softplus layer. This model is designed to predict brain age for both the whole brain regions and specific subregions, including the frontal lobe, central region, parietal lobe, occipital lobe, and temporal lobe. These predictions are then compared with chronological age.

We estimated BA from EEG data using a time-frequency representation of EEG as inputs and a deep neural network with a triangular filter, several GRU (Gated Recurrent Unit) layers, and an attention module. Figure 2 illustrates the architecture of the Auto-EEG-Brain AGE prediction model. The model includes a trainable triangular filter layer, two unidirectional GRU layers, an attention layer, and a Softplus layer. The predicted BA is the model output. The model utilizes the time-frequency graph as its input. The size of the input dimensions is [𝑁, 𝐶, 𝐹, 𝑇], where *N*, *C*, *F*, and *T* are the batch size, the number of channels (*C* = 19), frequency dimension of the time-frequency images (*F* = 157), and the number of segments in the time dimension of the time-frequency graph (*T* = 149), respectively.

The triangular filter layer aims to reduce dimensionality along the frequency axis of time-frequency graph features. Consequently, it transforms the input time-frequency graph $S_{i}$ into the output $x_{i}$ (Equation 1a), where $S_{i}\in {\mathbb{R}}^{F\times T\times C}$ and $x_{i}\in {\mathbb{R}}^{M\times T\times C}$ , with *M* < *F*. Here i∈[1,n] serves as the index of the time-frequency graphs. The trainable triangular filters are represented by $W_{f}\in {\mathbb{R}}^{F\times M}$ (Equation 1b). $f_{+}$ is an element-wise non-negative function that converts the trainable parameter matrix $W\in {\mathbb{R}}^{F\times M}$ into a non-negative matrix, utilizing the sigmoid function. $W_{L}\in {\mathbb{R}}^{F\times M}$ serves as a non-negative constant matrix, constraining the filter within a finite bandwidth. Inspired by Mel filter design, $W_{L}$ exhibits smaller bandwidths in low-frequency regions and larger bandwidths in high-frequency regions. Equation 2a converts the frequency $f\in \left[f_{\min},f_{\max}\right]$ to the logarithmic space $F_{\log}$ within the range $\left[F_{\log}^{\min},F_{\log}^{\max}\right]$ . Subsequently, the interval $\left[F_{\log}^{\min},F_{\log}^{\max}\right]$ is uniformly divided into F+1 sub-intervals to obtain $F_{\log}^{j}$ (as defined in Equation 2b). The elements of matrix $W_{L}$ , denoted as $W_{L}\left(m,k\right)$ , were satisfied according to Equation 2d, where m∈{1,2,…F} , k∈{0,1,2,…M−1} The function *f*(m), as defined in Equation 2C, is used therein.


(1a) $$x_{i}=W_{f}^{T}S_{i}$$

(1b) $$W_{f}=f_{+}\left(W\right)⊙W_{L}$$

(2a) $$F_{\log}=26.0*\log_{10}\left(1+\frac{f}{7.0}\right)$$

(2b) $$F_{\log}^{j}=F_{\log}^{\min}+j*\frac{F_{\log}^{\max}-F_{\log}^{\min}}{F+1},j\in \left[0,F+1\right]$$

(2c) $$f\left(j\right)=7.0*\left(10^{\frac{F_{\log}^{j}}{26.0}}-1.0\right)$$

(2d) $$W_{L}\left(m,k\right)=\left\{\begin{array}{c} 0,k<f\left(m-1\right)\\ \frac{k-f\left(m-1\right)}{f\left(m\right)-f\left(m-1\right)},f\left(m-1\right)\leq k<f\left(m\right)\\ 1,k=f\left(m\right)\;\\ \frac{f\left(m+1\right)-k}{f\left(m+1\right)-f\left(m\right)},f\left(m\right)<k\leq f\left(m+1\right)\;\\ 0,k>f\left(m+1\right) \end{array}\right.$$

The GRU layer, configured as a unidirectional architecture with $L_{1}$ layers and a hidden layer size of $H_{1}=64$ , encodes the temporal information within EEG signals, yielding an output tensor $a\in {\mathbb{R}}^{H_{1}\times n\times T}$ . Following the encoding process, the attention mechanism assigns weights to the output tensor $b\in {\mathbb{R}}^{H_{1}\times n}$ (defined by Equations 3a–3c), where $W_{\mathrm{att}}^{T}$ represents a trainable parameter matrix. Additionally, the Softplus activation function, described in Equation 4, ensures that the age output remains non-negative. The integration of these layers facilitates the model’s ability to encode input data effectively and automatically prioritize the most relevant segments based on task requirements, thereby enhancing both the interpretability and performance of the model.


(3a) $$b_{i}=\sum_{j=1}^{T}\rho_{ij}a_{ij}$$

(3b) $$\rho_{ij}=\frac{e^{f\left(a_{ij}\right)}}{\sum_{j=1}^{T}e^{f\left(a_{ij}\right)}}$$

(3c) $$f\left(a_{i}\right)=\tanh\left(W_{\mathrm{att}}^{T}a_{i}\right)$$

(4) $$z=\log\left(1+e^{x}\right)$$

### Training experiments: selection of models

**Figure 3:**
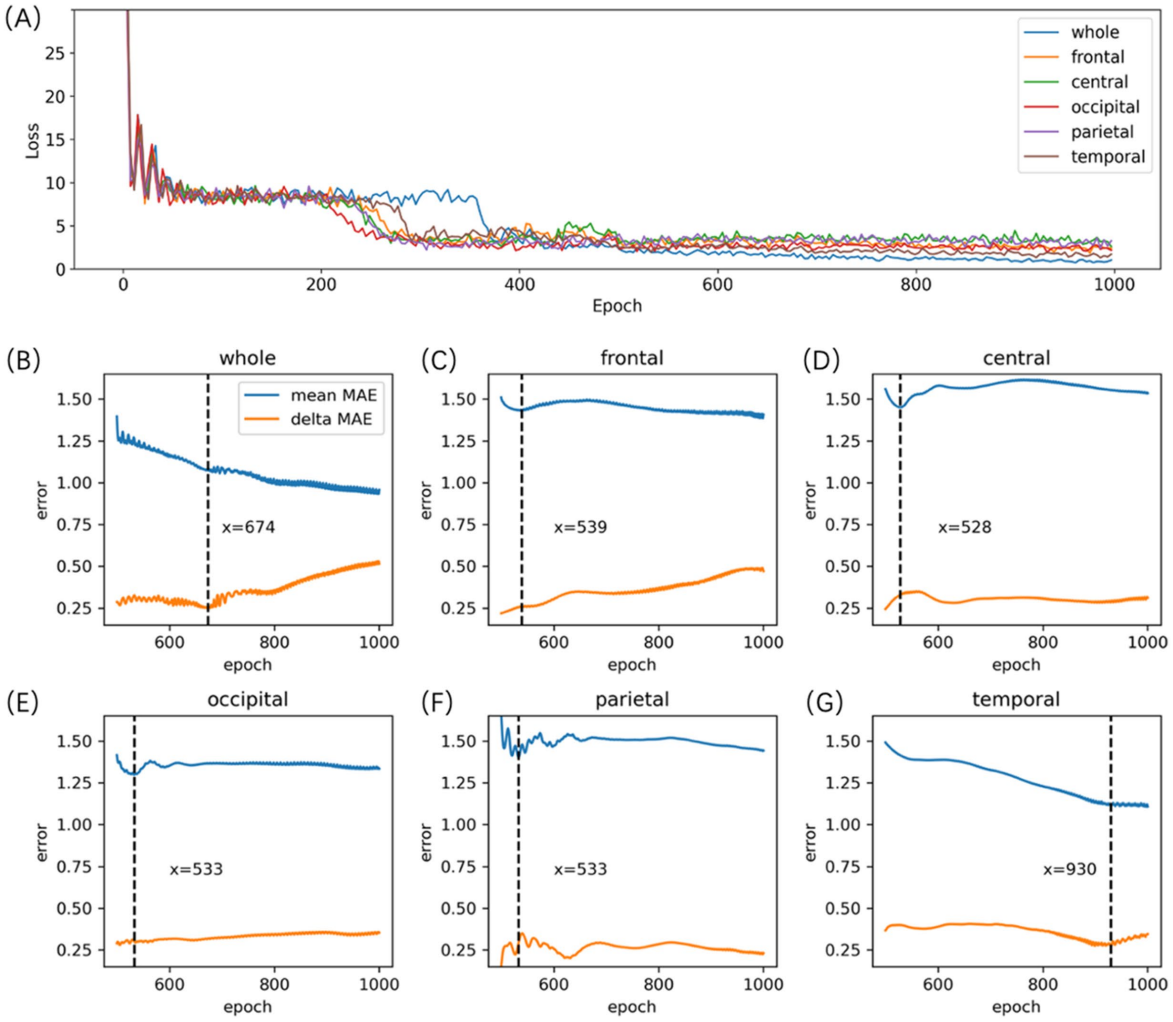
Auto-EEG-Brain AGE model selection. **(A)** the Loss curve of the Auto-EEG-Brain AGE model. **(B-G)** the MAE curves of the model for the whole brain, frontal, central, occipital, parietal, and temporal regions across both training and validation datasets. The blue line represents the curve of the MAE averaged across both the training and validation sets (mean MAE), whereas the orange line illustrates the curve representing the absolute values of the discrepancies in MAE observed between the training and validation sets (delta MAE). **(B-G)** indicate that the model for the whole brain, frontal, central, occipital, parietal, and temporal region reached their peak performance at the 674th, 539th, 528th, 533th, 533th, and 930th epochs, respectively.

Models were selected based on mean absolute errors (MAE) and deltaMAE (please refer to the Supplementary material for additional details regarding the results of the hyperparameter screening). meanMAE is calculated as the average of ${\mathrm{MAE}}_{\mathrm{train}}$ and ${\mathrm{MAE}}_{\mathrm{validate}}$ , while deltaMAE is the absolute value of the difference between ${\mathrm{MAE}}_{\mathrm{train}}$ and ${\mathrm{MAE}}_{\mathrm{validate}}$ . A lower value of meanMAE suggests better overall performance of the model across both training and validation datasets but may also indicate potential overfitting. Therefore, considering deltaMAE is crucial for assessing the model’s generalization ability. A smaller deltaMAE suggests greater consistency in the model’s performance between the training and validation datasets. The preferred model selection criterion involves selecting the one with the lowest meanMAE , provided that the deltaMAE remains relatively small. Figure 3A depicts the process of selecting the Auto-EEG-Brain AGE prediction model. Optimal performance for models across specific brain regions was achieved at different epochs as demonstrated in Figures 3B–G. To maximize data utilization and bolster the stability of prediction outcomes, the trained model was subsequently deployed to predict the BA for all qualifying 5-min awake EEG segments. The final BA estimation was determined by selecting the median of these BA predictions across segments.

## Results

### Correlations between CA and BA

**Figure 4:**
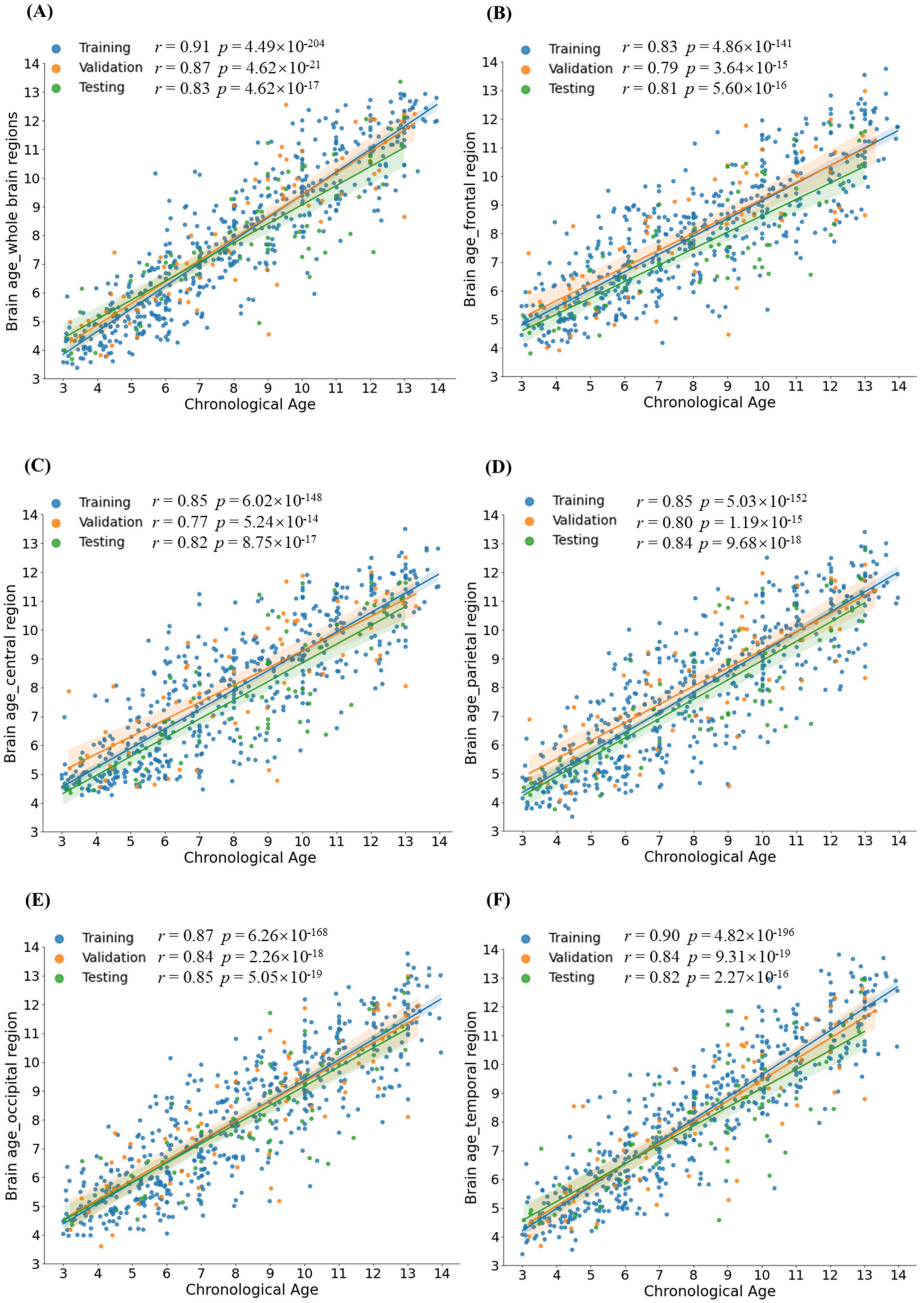
Group comparison of Brain AGE. **(A-F)** demonstrated statistical differences between the ASD group and the healthy group for different brain regions: the whole brain, frontal lobe, central region, parietal lobe, occipital lobe, and temporal lobe. Brain AGE of the ASD group significantly differed from the healthy group in various brain regions, indicating that Brain AGE can provide a biomarker for the evaluation of ASD. Brain AGE: Brain age gap estimate.

Pearson’s correlations were conducted between CA and BA in healthy children and ASD patients to evaluate the performance of the BA model. Significant correlations between CA and BA were observed in healthy children across both the whole brain and five distinct brain regions (with correlation coefficients *r* > 0.81 and *p*-values <0.001 for all examined regions, see Figure 4). In the testing set, Pearson’s correlations, along with their corresponding coefficient of determination (*R*^2^) and MAE, as depicted in Table 1. The MAE for both the whole brain and individual brain regions were less than 1.5 years, demonstrating the model’s robust predictive accuracy. Among ASD patients, significant Pearson correlation coefficients were observed between CA and BA across different brain regions, as depicted in Table 1. However, in individuals with ASD, the correlation between BA and CA reached a maximum of 0.76 in whole-brain age prediction, while regional brain age predictions showed BA-CA correlations around 0.7, all significantly lower than those observed in healthy children (*p*-values <0.004).

**Table 1:** Correlations between CA and BA among healthy children and ASD patients.

Brain regions	Healthy children				ASD	
	*r*	*p*	*R*^2^	MAE (years)	*r*	*p*
Whole brain regions	0.83	4.62 × 10^−17^	0.67	1.31	0.76	3.30 × 10^−20^
Frontal lobe	0.81	5.60 × 10^−16^	0.58	1.49	0.69	9.61 × 10^−15^
Central lobe	0.82	8.75 × 10^−17^	0.62	1.48	0.66	9.31 × 10^−14^
Parietal lobe	0.84	9.68 × 10^−18^	0.66	1.42	0.70	5.99 × 10^−16^
Temporal lobe	0.82	2.27 × 10^−16^	0.65	1.30	0.68	2.30 × 10^−14^
Occipital lobe	0.85	5.05 × 10^−19^	0.71	1.26	0.69	5.15 × 10^−15^

### Analysis of brain AGE for ASD patients

To investigate the clinical applicability of brain age prediction models in individuals with ASD, a paired sample *t*-test was employed to examine potential discrepancies between BA values and CA values in autism patients. In individuals with ASD, BA was significantly elevated compared to CA (*p*-values < 0.001), suggesting potential abnormalities in functional neurodevelopment underlying ASD pathophysiology (refer to Table 2 for further details).

**Table 2:** Results of a paired sample t-test between BA and CA in autism patients.

Brain regions	Mean _BA-CA_ (SD)	*t*	*p*	*d*
Whole brain regions	0.76 (1.13)	5.72	1.14 × 10^−7^	0.40
Frontal lobe	1.18 (1.49)	7.81	6.51 × 10^−12^	0.64
Central lobe	1.04 (1.57)	6.57	2.48 × 10^−9^	0.55
Parietal lobe	0.75 (1.45)	5.14	1.43 × 10^−6^	0.40
Temporal lobe	0.64 (1.46)	4.33	3.50 × 10^−5^	0.35
Occipital lobe	1.07 (1.53)	6.88	5.89 × 10^−10^	0.56

Meanwhile, a repeated measures ANOVA was performed to access differences in Brain AGE across brain regions. A significant main effect of region was found, *F* (4, 388) = 7.72, *p* < 0.001, *η*_p_^2^ = 0.018 (see Supplementary Figure S4). Bonferroni-adjusted post-hoc comparisons revealed that Brain AGE was significantly higher in the frontal lobe (*p* = 0.0006), temporal lobe (*p* = 0.0162), and central lobe (*p* = 0.0012) compared to the occipital lobe. Moreover, Brain AGE in the frontal (*p* = 0.0027) and central lobes (*p* = 0.0012) was significantly higher than in the parietal lobe. No other pairwise comparisons reached statistical significance (all *p*-values were greater than 0.1993).

### Comparison of brain AGE between the healthy group and the ASD group

**Figure 5:**
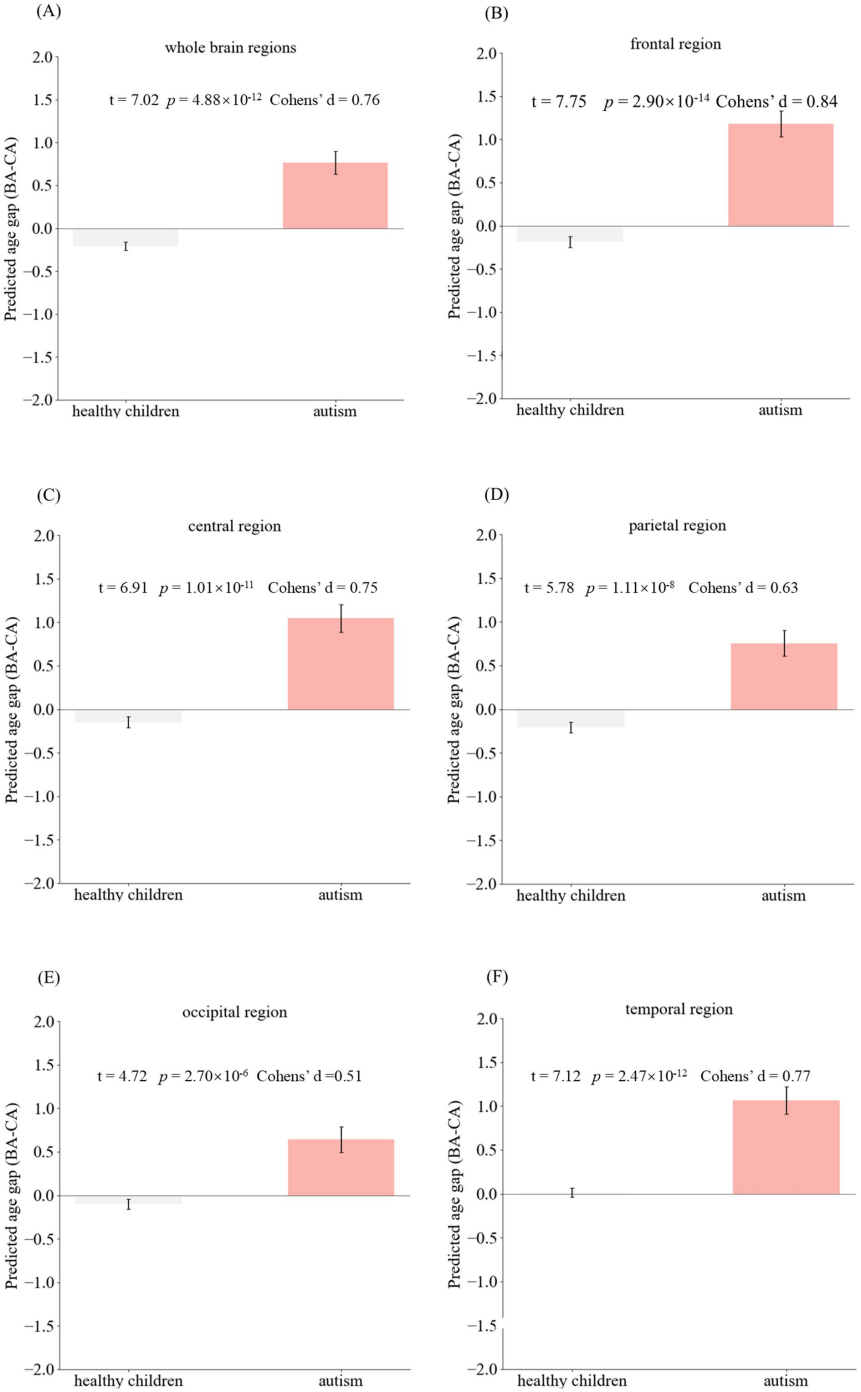
Scatter plots depict the relationship between CA and predicted BA. Each point corresponds to one participant. Panels **(A-F)** illustrate the correlations between CA and BA for different brain regions: the whole brain, frontal lobe, central lobe, parietal lobe, occipital lobe, and temporal lobe, respectively. The significant correlations observed between CA and BA in both the testing and validation sets suggest that the Auto-EEG-Brain AGE model accurately captures the underlying characteristics of brain aging.

An independent-sample *t*-test showed that ASD patients exhibited higher values of Brain AGE than healthy children in all brain regions, including the whole brain, frontal lobe, central lobe, temporal lobe, parietal lobe, and occipital lobe (*p*-values <2 × 10^−6^, see Figure 5). For comparability, 98 healthy individuals were randomly selected and matched with ASD patients based on their age and gender distribution. The results of the independent sample *t*-test can be found in Supplementary Figure S4.

## Discussion

In this study, we introduced the Auto-EEG-Brain AGE prediction model, which uses EEG data and deep learning to estimate brain age in children. The model processes time-frequency representations through trainable triangular filters to focus on relevant frequency bands, encodes temporal features with a GRU-based model, and applies a self-attention mechanism to highlight age-relevant features. The model showed strong performance in typically developing children, with correlation coefficients between predicted and chronological age exceeding 0.8 for whole-brain and regional analyses. However, in children with autism spectrum disorder (ASD), these correlations were lower (0.66–0.76), and the Brain AGE was significantly higher across all brain regions compared to typically developing children. These results indicate that our model and the Brain AGE metric effectively capture and reflect atypical brain development in ASD, highlighting the potential of EEG-based deep learning models for early identification and monitoring of neurodevelopmental disorders.

In recent years, numerous studies have explored brain age prediction using EEG signals, yet most investigations have primarily focused on adult populations. Moguilner et al. (11) proposed the concept of a “brain clock” to assess aging and dementia variations across geographical populations, developing a deep learning model architecture based on EEG data that achieved a root mean square error (RMSE) of 6.45 years in age prediction for subjects aged 21–92 years. Al Zoubi et al. (12) extracted spectral and connectivity features from EEG signals and employed machine learning models for brain age estimation, reporting a mean absolute error (MAE) of 6.87 years and a correlation coefficient (*r*) of 0.6 in a dataset with a mean age of 34.8 years. Sun et al. (13) estimated brain age through sleep EEG recordings across two datasets (age ranges 18–80 and 40–80 years respectively), achieving a prediction MAE of 7.6 years. Additional research has addressed neonatal brain age prediction, with Davoudi et al. (14) applying deep neural networks to EEG analysis for estimating brain age in infants aged 3–14 months, achieving prediction errors of approximately 1 month. However, current research lacks investigations into brain age prediction for developmental children aged 3–18 years. Our study aims to address this critical gap by developing an assessment framework to evaluate neurodevelopmental patterns in children during this crucial growth period.

The prevalence of ASD has been on the rise in recent years. In the United States, the prevalence of ASD increased to 2.0–7.0 per 1,000 in the 1990s; while the prevalence became 1/54 in 2016 and 16.8 per 1,000 in 2018, according to the data from the Centers for Disease Control and Prevention (CDC) (15). Previous studies have elucidated the abnormal brain development in ASD from different perspectives, including structure and function. In terms of anatomical structure, it has been found that infants and children with ASD exhibit early brain overdevelopment in cellular distribution (16, 17), presenting with an excessive number of prefrontal cortical nerves (17). Previous longitudinal and cross-sectional magnetic resonance imaging studies reported age-specific anatomical abnormalities in patients with ASD. They found that ASD overgrows in early life but declines at an accelerated rate in adolescence and early adulthood. Specifically, in ASD, the frontal lobes show the most severe enlargement at 2–3 years of age, and frontal gray matter in children with ASD has an abnormal growth rate. Neuroimaging studies of patients with ASD have revealed abnormalities in the internal connectivity of the brain (18, 19), with neurons in prefrontal and temporal lobe regions having reduced functional connectivity with other brain regions (20). Hypoplasia of the cerebral corpus callosum has also been found in ASD (21, 22).

In recent years, with the advancement of EEG technology and the development of the field of artificial intelligence, electrophysiological studies on ASD have become increasingly progressive. In 2008, Coben et al. (23) analyzed the EEG characteristics of children with autism and found that a large number of theta oscillations appeared in the posterior part of the brain and sparse *δ* oscillations appeared in the frontal lobe of ASD; furthermore, the neural connectivity of the brain was abnormal in the children with autism. Catarino et al. (24) analyzed EEG data in autism using a multi-scale entropy approach. It showed abnormal and complex EEG characteristics in both the temporal–parietal and occipital lobes. Duffy and Als (25) found a stable pattern of EEG spectral coupling through a study with a large number of subjects, which was effective in identifying children with autism. Sheikhani et al. (26) used quantitative EEG to detect the abnormalities of autistic children’s features. They found lower spectral energy in the left brain region and higher connectivity in the temporal lobe gamma (36–44 Hz) frequency band in children with autism. Wang et al. (27) pointed out in a study about EEG of ASD patients that the electrophysiological power changes in ASD patients in the resting state were in the form of a U-shape, i.e., excessive power in the low-and high-frequency bands, abnormal functional connectivity, and power enhancement in the left hemisphere of the brain. The EEG characteristics of ASD patients are relatively stable for a short period (28). Coben et al. (23) suggested that autistic patients in the age group of 6 to 11 years old showed dysfunction and incomplete activation of neurological functions in frontal and posterior brain regions, suggesting dysfunction of integration of the frontal lobe and posterior brain regions and abnormal neural connectivity, as shown in the analysis of quantitative EEG. Therefore, EEG plays a crucial role in the early diagnosis of ASD in childhood.

Based on the above theoretical foundation, we constructed an Auto-EEG-Brain AGE prediction model based on the neural network method of GRU with a total of 772 normal EEG data from 659 healthy children aged 3–14 years and validated the model. Using time-frequency graphs, the model inputs can enhance the capture of the time-variant multi-band information from EEG signals. Within the model, trainable triangular filters perform weighted summation across different frequency bands. Compared with wavelet transform, combining STFT for calculating time-frequency graphs with trainable triangular filters improves computational efficiency. On the other hand, the trainable weighting coefficients for frequency bands allow the model to focus more on age-related features within the time-frequency graphs. The GRU layers can capture the temporal information in the time-frequency graphs. The self-attention layer integrates the temporal outputs from the preceding layer, thereby focusing the features more effectively. Lastly, the Softplus layer ensures that the predicted BA output is positive. The healthy children’s EEG data calculated the *r*-value, MAE, and *R*^2^ of the whole brain and each brain region (frontal, central, parietal, temporal, and occipital regions). The *r*-value of the model at the whole-brain level reached 0.8 or above, regardless of the training set, validation set, or test set, and the highest value was up to 0.91, which indicated that the BA predicted by the model had correlated extremely well with the CA. The best *r*-value of sub-brain regions was in the occipital lobe (*r* = 0.9), suggesting that the predicted BA of the model for the occipital lobe has a higher correlation than the models for other brain regions. The MAE of the model for predicting the BA reached 1.32 years at the whole-brain level, and the MAE of the model for the sub-brain regions ranged from 1.35 to 1.53 years, with the best prediction of the model also for the occipital lobe at the level of 1.35 years. The *R*^2^ of the model in the occipital lobe reached 0.71, suggesting the BAs predicted by the model fit well with the actual ages. Compared with previous BA-related studies in the pediatric field, the BA prediction for healthy children by the model in this study is relatively more accurate. Ball et al. (29) reported a MAE of 1.54 years for their BA prediction model in a study involving 786 individuals aged 3–21 years. Khundrakpam et al. (30) achieved an MAE of 1.68 years for 308 typically developing children with a mean age of 12.9 ± 3.8 years. In conclusion, the Auto-EEG-Brain AGE prediction model is highly reliable which can accurately predict the BA of healthy children aged 3–14 years in the whole brain region and at the level of all brain regions (especially at the level of the occipital lobe), accurately reflecting the individual’s full age and evaluating the brain development.

After validating the predictive performance of the Auto-EEG-Brain AGE prediction model in healthy children, we analyzed the EEG data of the 98 ASD patients. The correlation between predicted BA and actual age, as well as the correlation values at the whole brain level and each brain region in the ASD group, were obtained. We compared the data of patients with ASD and that of the healthy group. *r*-values of the ASD group amounted to 0.76 at the level of the whole brain and ranged from 0.66–0.7 at the level of the sub-brain regions, with the parietal region being the best at 0.7, suggesting that there is still a correlation between the predictive value of BA in the ASD group and their actual age. Comparative analysis of the *r*-values of healthy children and children with ASD at the same level suggests that the *r*-values of the healthy group were higher than those of the children in the ASD group, both at the level of the whole brain and at the level of each brain region (whole brain 0.91 vs. 0.76, frontal lobe 0.83 vs. 0.688, central region 0.85 vs. 0.66, parietal lobe 0.85 vs. 0.7, occipital lobe 0.9 vs. 0.69, temporal lobe area 0.87 vs. 0.688), suggesting that the relevance of BA prediction was significantly lower in the ASD group than in the healthy group. The diminished correlation of BA prediction in the ASD group may reflect genuine neurodevelopmental differences; that is, the brain development of ASD patients has its unique developmental characteristics under the influence of the framework of normal brain developmental laws. However, it may also indicate limitations in the generalizability of the prediction model, which requires further investigation.

BA and Brain AGE are objective tools that can be used to evaluate individualized brain development and can be used as objective biomarkers for neurodevelopmental disorders. In the study related to the use of BA in the assessment and diagnosis of ASD, the statistical analysis suggests that there is a significant deviation between the predicted BA and its full age in the ASD group. The mean values of the BA gaps in the whole brain, frontal lobe, central, parietal lobe, occipital lobe, and temporal lobe regions are 0.76, 1.18, 1.04, 0.75, 0.64, and 1.07 years old, respectively. The predicted BA values of ASD patients were significantly higher than the full age in the whole brain and in each brain region, with the differences in the frontal, central, and temporal lobe regions being more significant than 1 year, suggesting that the predicted BA values can be used as an objective biomarker for the assessment of ASD and can help clinicians to make a diagnosis of the disease. Further, the study of the difference between the predicted BA difference of each brain region suggested that the predicted BA difference was significantly different among brain regions, i.e., the predicted BA difference of frontal (*p* = 0.0006), temporal (*p* = 0.0162), and central (*p* = 0.0012) regions was significantly greater than that of occipital regions. The predicted BA difference among frontal, temporal, and central regions had no significant difference (*p*-values = 1), nor was there a significant difference in predicted BA between occipital and parietal regions (*p* = 1). This study provides a theoretical basis for studying the brain developmental characteristics of ASD in different brain regions and refining the assessment and diagnostic indexes of ASD in different brain regions.

For ASD, positive Brain AGE values indicate advanced structural maturation of the brain, while negative values indicate delayed structural maturation. In addition, it has been demonstrated that Brain AGE correlates with the severity of ASD, especially in terms of communication and social interaction skills (31), which can be used in the assessment of the condition and prognosis of ASD. Statistical analysis of Brain AGE suggested that Brain AGE in the ASD group was significantly greater than that in the healthy group (*p*-values <2 × 10^−6^) at the level of the whole brain and in each brain region. Therefore, brain AGE has a potential role in the diagnosis of ASD.

### Strengths and limitations

We constructed a BA prediction model based on EEG data and demonstrated that the model could predict the BA of healthy children and children with ASD with accuracy at the level of the whole brain and the level of each brain region. By pairwise analysis, it was suggested that the brain development of ASD children still followed the normal physiological developmental pattern, but the correlation was weakened. The model could accurately identify brain development and may be used as a biomarker to assess ASD and assist clinicians in diagnosing it. Moreover, compared with previous BA prediction models primarily based on neuroimaging data such as sMRI (2, 30) and fMRI (32), the present study employs EEG signals as the analytical indicator, which is more suitable for the pediatric population. It has higher sensitivity and lower cost, and is more conducive to future promotion and popularization.

As for limitations, first, the age distribution of children in the healthy group is not yet balanced, mainly distributed from 3 to 14 years old, which is not enough to construct the curve of BA change with full age for children of all age groups. Second, only 98 patients were included in the ASD group, which is a relatively small sample size. More independent datasets of ASD children cases are still needed in the future for statistical analysis to confirm the correlation and for gender grouping to observe the gender-related differences in BA for ASD. Finally, this study is a cross-sectional study, and the patients in the ASD group with different disease stages and intervention bases, which may result in bias. We plan to incorporate ASD-related assessment scales, such as the ADOS-2, Vineland-3, and Social Responsiveness Scale-2 (SRS-2), to conduct group analyses and prospective longitudinal studies. Additionally, we intend to integrate our EEG-based model with imaging-based BA prediction models (e.g., those using sMRI and fMRI) to perform multimodal BA analysis. This multimodal approach will allow us to predict children’s BA from different dimensions, thereby enhancing the predictive efficacy of the model.

## Conclusion

The BA and Brain AGE can be applied to analyze cognitive behaviors and functions for children with ASD comprehensively. Besides the value of BA and Brain AGE in the diagnosis of ASD, the evaluation for the effect of intervention and prognosis will be comprehensively evaluated. The analysis made by BA and Brain AGE, combined with multiple related aspects of the disease (e.g., brain imaging, cognitive behavior, genes, etc.), can bring a more comprehensive and profound explanation of brain developmental abnormalities in ASD.

## Data Availability

The original contributions presented in the study are included in the article/Supplementary material, further inquiries can be directed to the corresponding authors.
