# Supplementary material for: Brain age prediction model based on electroencephalogram signal and its application in children with autism spectrum disorders
Source: Front Neurol. 2025 Jun 18;16:1605291. doi: 10.3389/fneur.2025.1605291 (PMC12213353; doi:10.3389/fneur.2025.1605291)
Supplement: Supplementary file 1 [file Supplementary_file_1.docx]

Supplemental materials

Hyperparameter screening results

There are two hyperparameters for data augmentation during the neural network training process: $\alpha$, which is applied for random perturbation of $Y_{i}$, and $\beta$ for random scaling of $Z_{i}^{j}$. Additionally, there is another hyperparameter $\gamma$, denoting the artifact ratio in the segments. The method of controlled variables was conducted for the selection of the hyperparameters $\alpha$, $\beta$, and $\gamma$. The training results demonstrated that the model attains relative optimality when the hyperparameters are set at $\alpha=0.1$, $\beta=0.05$ and $\gamma=0.25$ (Details of the hyperparameter selection can be found in Appendix Figure S1, S2, S3).

Under the conditions with hyperparameters set at $\alpha=0.1$, $\beta=0.05$ and $\gamma=0.25$, models were independently trained for the whole brain, the frontal, central, occipital, parietal, and temporal regions. These models started to progressively converge from the 550th epoch onwards. To select an optimal model, we investigated the changes in mean absolute errors (MAE) on the training set (${MAE}_{\mathrm{train}}$) and the validation set (${MAE}_{\mathrm{validate}}$) for each model from the 500th to 1000th epoch. $mean MAE$ and $delta MAE$ were computed for models evaluation.


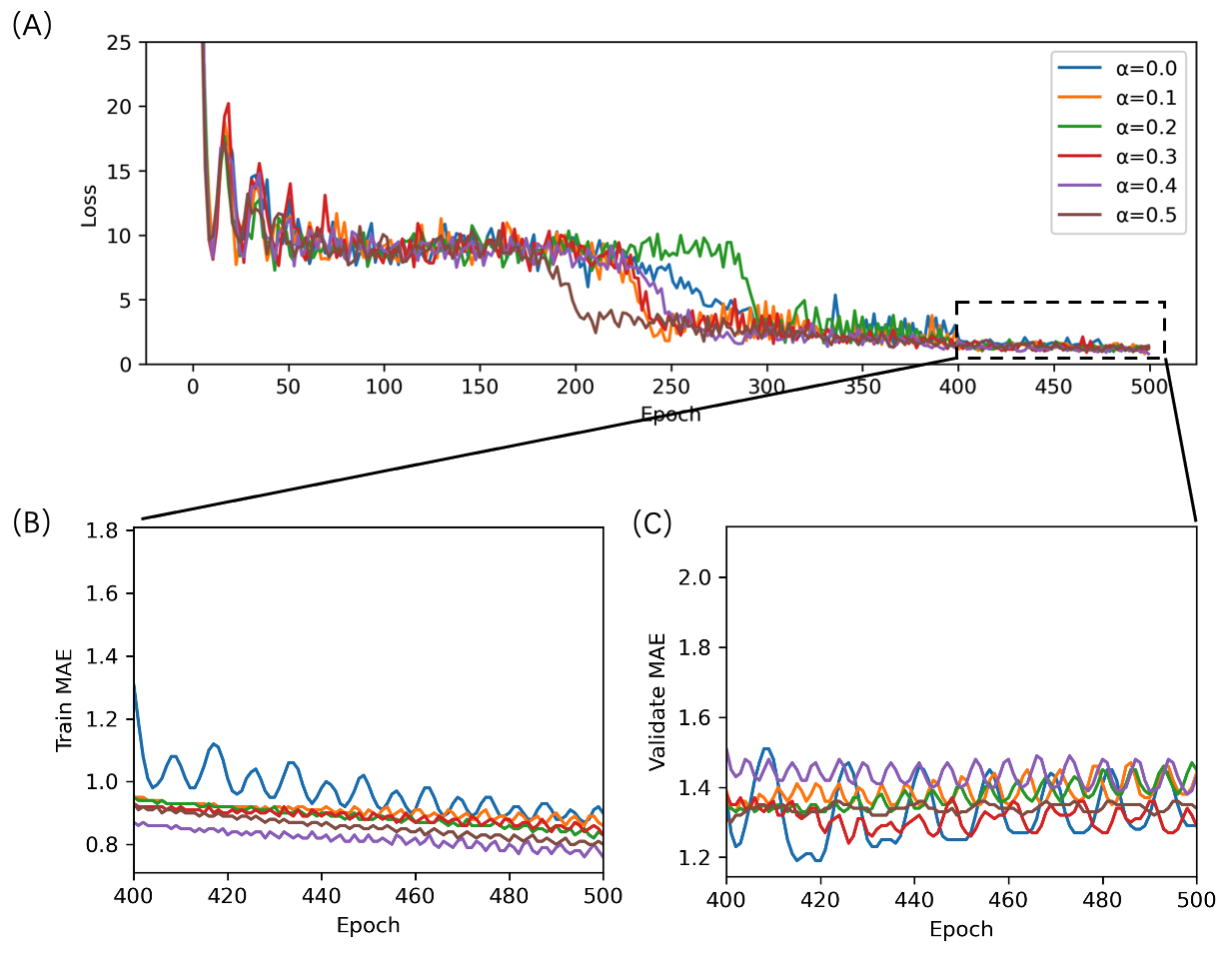


Figure S1. Experiment on Hyperparameter α Selection. (A) Training Loss curves at different α values; (B) and (C) are the MAE variation curves on the training set and validation set, respectively, during 400-500 epochs


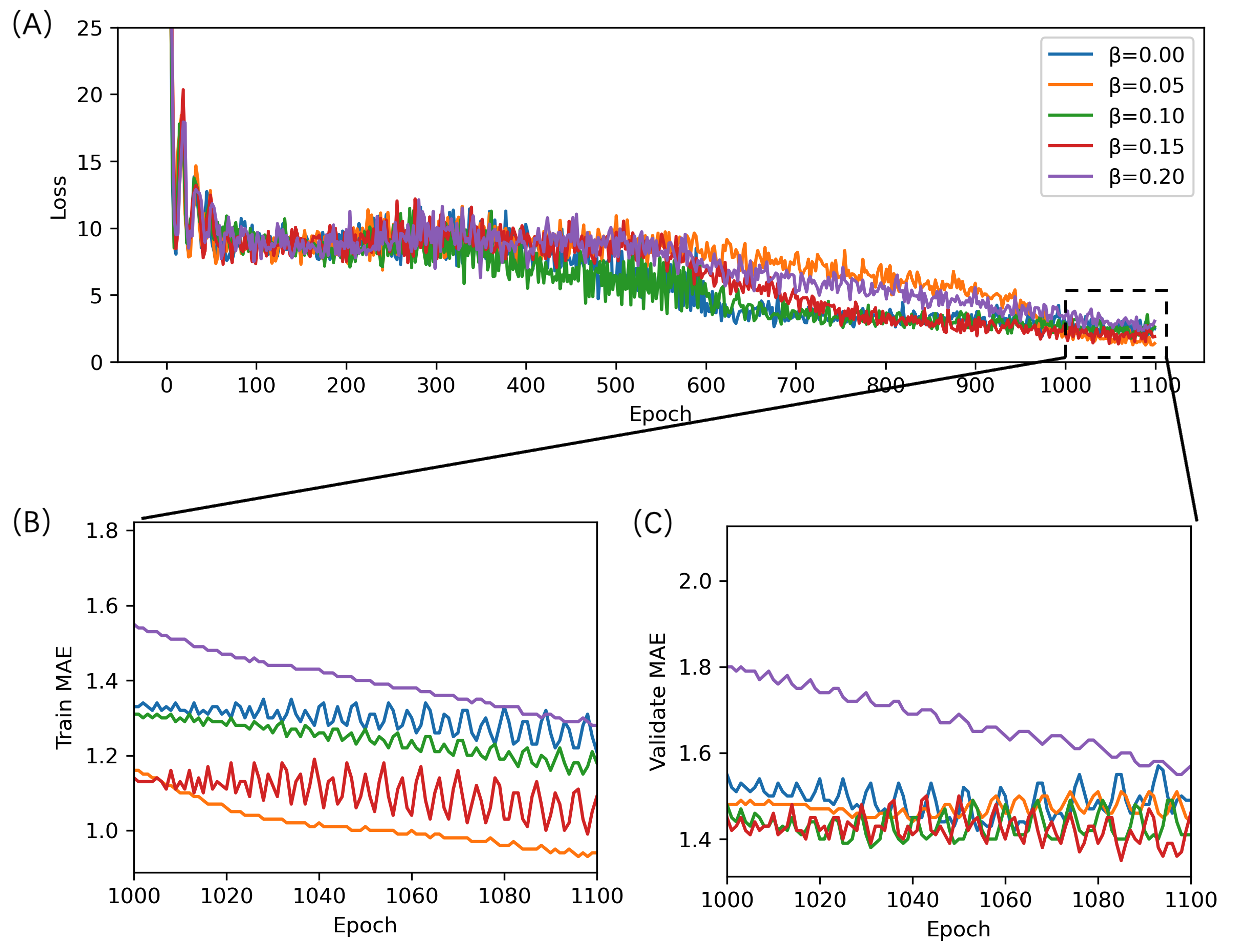


Figure S2. Experiment on Hyperparameter β Selection. (A) Training Loss curves at different β values; (B) and (C) are the MAE variation curves on the training set and validation set, respectively, during 1000-1100 epochs.

*
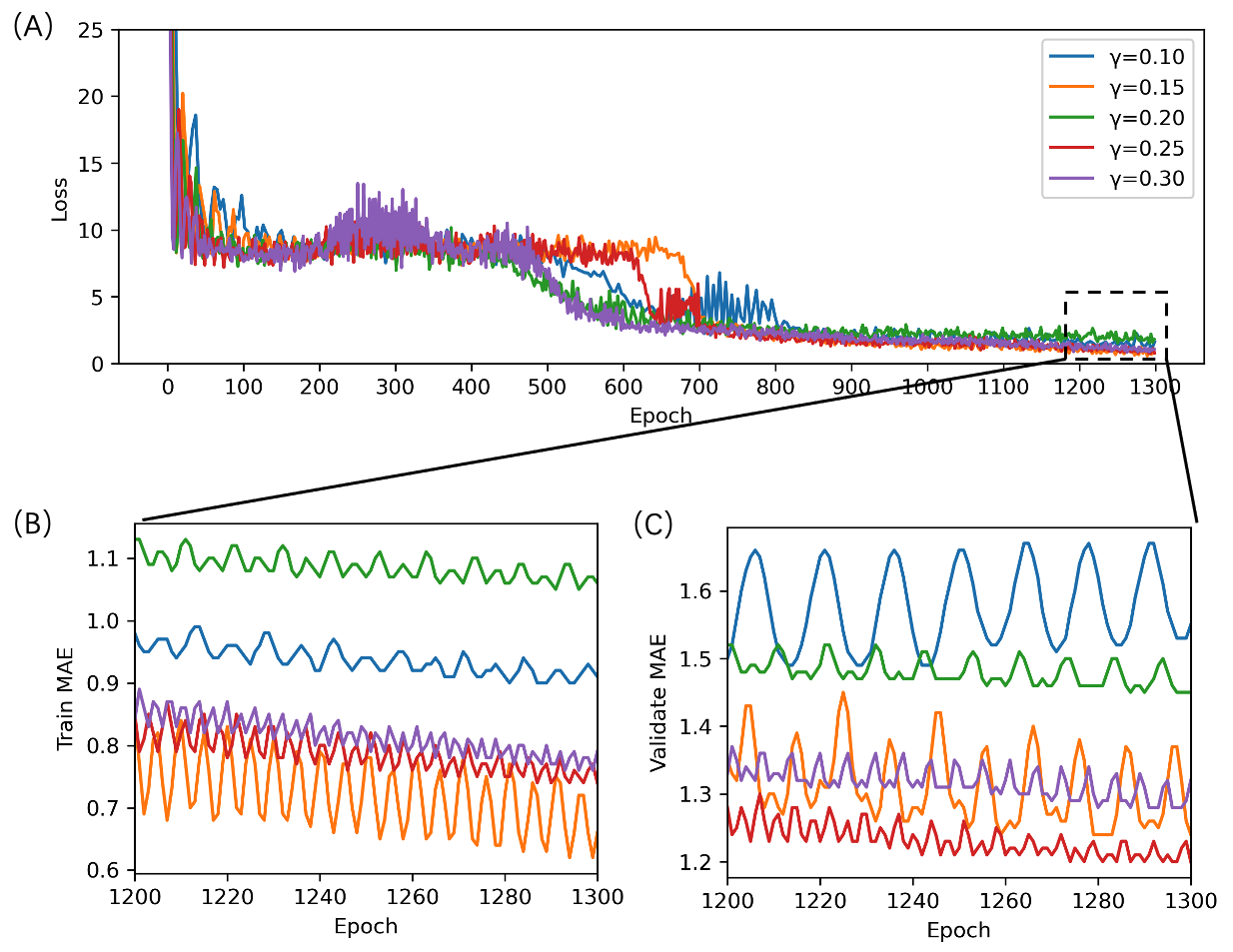
*

Figure S3. Experiment on Hyperparameter γ Selection. (A) Training Loss curves at different γ values; (B) and (C) are the MAE variation curves on the training set and validation set, respectively, during 1200-1300 epochs.


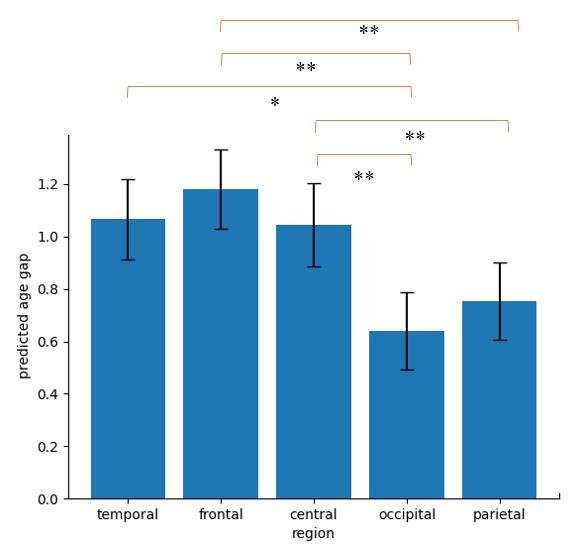


Figure S4. Results of the One-Way Repeated Measures ANOVA across different brain regions for ASD patients. *** p < 0.001, ** p < 0.01, * p < 0.05.


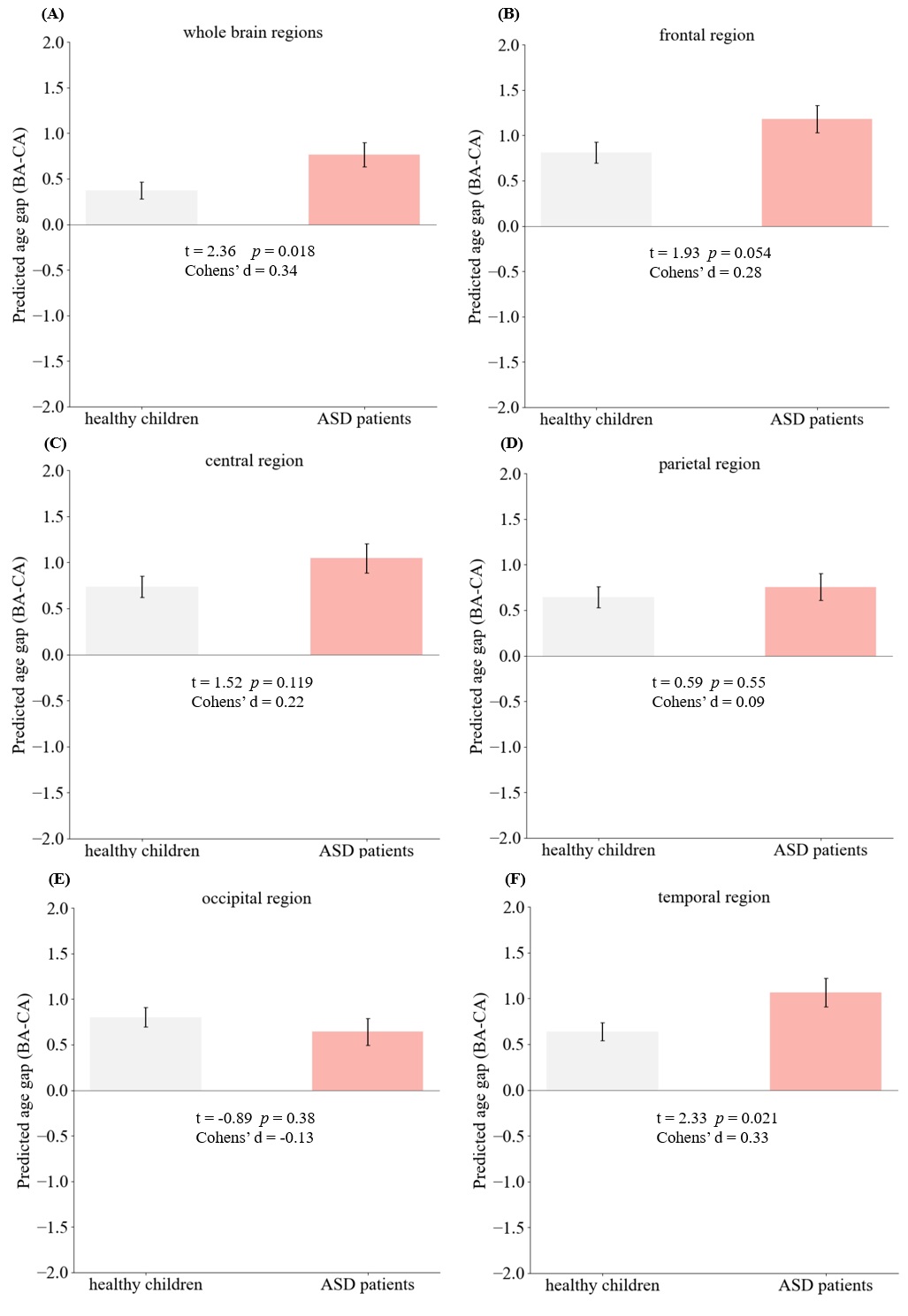


Figure S5. Comparison of Brain AGE for matched healthy children and individuals diagnosed with ASD.
